# Hypoactivity Affects IGF-1 Level and PI3K/AKT Signaling Pathway in Cerebral Structures Implied in Motor Control

**DOI:** 10.1371/journal.pone.0107631

**Published:** 2014-09-16

**Authors:** Julien Mysoet, Marie-Hélène Canu, Caroline Cieniewski-Bernard, Bruno Bastide, Erwan Dupont

**Affiliations:** 1 Université Lille Nord de France, Lille, France; 2 « Physical Activity, Muscle and Health » laboratory, EA 4488, IFR 114, Université Lille 1, Sciences et Technologies, Villeneuve d'Ascq, France; University of Louisville, United States of America

## Abstract

A chronic reduction in neuromuscular activity through prolonged body immobilization in human alters motor task performance through a combination of peripheral and central factors. Studies performed in a rat model of sensorimotor restriction have shown functional and biochemical changes in sensorimotor cortex. However, the underlying mechanisms are still unclear. Interest was turned towards a possible implication of Insulin-like Growth Factor 1 (IGF-1), a growth factor known to mediate neuronal excitability and synaptic plasticity by inducing phosphorylation cascades which include the PI3K–AKT pathway. In order to better understand the influence of IGF-1 in cortical plasticity in rats submitted to a sensorimotor restriction, we analyzed the effect of hindlimb unloading on IGF-1 and its main molecular pathway in structures implied in motor control (sensorimotor cortex, striatum, cerebellum). IGF-1 level was determined by ELISA, and phosphorylation of its receptor and proteins of the PI3K–AKT pathway by immunoblot. In the sensorimotor cortex, our results indicate that HU induces a decrease in IGF-1 level; this alteration is associated to a decrease in activation of PI3K-AKT pathway. The same effect was observed in the striatum, although to a lower extent. No variation was noticed in the cerebellum. These results suggest that IGF-1 might contribute to cortical and striatal plasticity induced by a chronic sensorimotor restriction.

## Introduction

It is admitted since a very long time that regular physical exercise is good for health. These last years, an increasing amount of studies has demonstrated that exercise promotes brain plasticity and has beneficial consequences on the brain, in young health population as well as during ageing [1]–[3]. Animal models of exercise suggest that training might enhance brain function through several mechanisms, such as neurogenesis, angiogenesis, synaptogenesis (for review, [4]) and increase in growth factors such as Brain Derived Nerve Factor (BDNF) and Insulin-like Growth Factor-1 (IGF-1) [1], [5]. In particular, the link between IGF-1 and exercise is well documented, and this molecule has been proposed as the mediator of exercise action on the brain.

At contrast with the numerous papers relating the relationships between physical activity and the brain, only a few reports concerned the effect of hypoactivity on the brain. Yet, a reduction of physical activity is commonly encountered by everyone throughout the life span, for instance during confinement to bed. Data performed by our team in the rat hindlimb unloading model (HU), a model of functional hypoactivity, have provided evidence for changes within the sensorimotor cortex. The excitability of pyramidal cells in the motor cortex is decreased [6], [7], and their dendritic spine density is increased [8]. Recent studies have suggested that alterations in corticospinal excitability should also be taken into account to explain the degradation of posture and locomotion in human following bed-rest [9], [10]. This hypothesis is sustained by data by our team which put forward the role of motor cortex in motor disabilities induced by HU in rodents [11].

Today, our interest is turned towards the role of IGF-1 in the neuronal plastic mechanisms induced by HU. IGF-1 is a polypeptide synthesized by many tissues, including the brain. Its concentration is higher during development, but this growth hormone is also present during adulthood. The bioavailability of IGF-1 is modulated by IGF binding proteins (IGFBPs) that bind IGF-1 with high affinity. The release of IGF-1 by IGFBPs is regulated through mechanisms such as phosphorylation or protease-mediated shedding. IGF-1 plays several important roles in the brain, where it modulates synaptic plasticity, neuronal excitability, and dendritic growth ([12] for review). The effects of IGF-1 are mainly mediated through IGF-1 receptors. Receptor activation in turn recruits intracellular cascades, which includes the phosphoinositide 3-kinase (PI3K)–AKT and in a lesser extent mitogen-activated protein kinase (MAPK) pathways [12]–[15].

Now, the question that arises is whether, in contrast with exercise, deleterious effects of HU on motor performance are due to decrease in brain IGF-1 content. In a first attempt to answer this question, we determined whether hypoactivity affects IGF-1 level, IGF-1 receptor expression and activation, and its main signaling pathway, i.e. PI3K/AKT, in various brain areas involved in motor control.

## Methods

### Ethics statement

All procedures described below were approved by both the Agricultural and Forest Ministry and the National Education Ministry (veterinary service of health and animal protection, authorization 59-00999). All efforts were made to minimize suffering.

### Animals and treatment

Adult male Wistar rats (280–320 g) were randomly divided into two groups: C (control) and HU14 (Hindlimb Unloading for 14 days). The animals were housed under temperature and light controlled conditions (23°C, 12-h light/12-h dark cycle). Rats were acclimated at least one week after their arrival to the animal facility. They were regularly handled. Both groups were housed in the same room, with the same conditions, and had *ad libitum* access to food and water.

Hindlimb unloading was performed using the tail suspension model [16]. The rat's tail was washed with soapy water, rinsed, passed successively with alcohol and ether, and coated with a solution of collodion 4% (Merck). Once dry, the tail was surrounded with a hypoallergenic adhesive tape (Elastoplast). This cast was secured to an overhead swivel that permitted 360° rotation. The height was adjusted so that the inclination of the rat body formed an angle of 30° with the horizontal. This situation prevented the contact of the hindlimbs with the ground, whereas the rats were allowed to move freely on their forelimbs. HU14 rats were placed next to the other; thus, they can have social interactions with their neighbors, whereas C rats were housed with 2 other rats.

### Tissue sampling

The blood and brain tissue samples were taken under deep anesthesia (sodium pentobarbital, 60 mg/kg) (Ceva Animal Health). Firstly, the thoracic cavity was open to allow access to the heart. A blood sample (1 ml) was obtained from the left ventricle. The blood was placed in a tube containing ethylenediaminetetraacetic acid (EDTA; Becton Dickinson) to prevent coagulation, and placed on ice. After centrifugation (3000×g for 15 min), plasma was collected and immersed in liquid nitrogen and stored at −80°C. Intracardiac infusion of ice-cold solution of phosphate buffered saline containing dextrose (5 mM) was then performed until total exsanguination of the animal. Then, the head of the animal was fixed in a stereotaxic frame, the skin incised and a craniotomy was performed to expose the cerebral cortex. The dura-mater was resected. A sample of sensorimotor cortex was taken at stereotaxic coordinates anterior 0 to −2 and lateral 2 to 4, corresponding to the display area of the hindlimb, and at coordinates anterior −5 to −7 and lateral 3 to 6, corresponding to visual cortex. Striatum and cerebellum were removed. The samples were placed in liquid nitrogen and stored at −80°C. The total duration of the sampling did not exceed 7 min.

### IGF-1 measurement

IGF-1 measurement was performed by an ELISA kit (Quantikine ELISA Mouse/Rat IGF-1, R&D Systems) on 14 rats (7 C and 7 HU). Brain samples were resuspended into an extraction buffer (15 µL of buffer/mg of tissue) developed by Adams et al. [17] [sodium acetate 7.5 mM; acetic acid 92.5 mM, pH 3.6; protease and phosphatase inhibitors (PhosSTOP phosphatase inhibitor and complete mini protease inhibitor cocktail tablets, Roche)]. The acidic pH separates IGF Binding Protein from IGF-1 and makes IGF-1 more accessible to antibodies. The samples were extracted using a potter-elvehjem homogenizer, and then homogenized for 1 h at 4°C. Samples were finally centrifuged at 3000×g for 10 min at 4°C. The supernatants were recovered and 250 µL of extraction buffer was added back to the pellet. The samples were homogenized, centrifuged, and the supernatants were again recovered. The two supernatants were mixed and the solution is concentrated by Vivaspin 500 (cutoff 3 kDa, Sartorius Stedim Biotech) at 13000×g for 90 min at 4°C. The samples were dried by SpeedVac Concentrator (Thermo Scientific) at 30°C for 1 h and then stored at −20°C.

Brain samples were diluted in 250 µL of 0.1 M HEPES buffer, pH 7.8, centrifuged for 10 min at 3000×g, the supernatants were collected and the pH is adjusted to 7. Plasma samples were diluted in the “Calibrator Diluent RD5–38” supplied in the kit. The measurement and the reagents were prepared according to the instructions given by the manufacturer's instructions.

### Pi3K-AKT signaling pathway analysis

Western blot analyses were performed on a total of 24 rats (n = 13 for C; n = 11 for HU14) in order to determine the activation status of several key signaling proteins such as IGF-1 receptor (n = 7–8 per group), Akt (n = 5–8 per group), GSK3β (n = 7–10 per group) and p70S6K (n = 4–7 per group) in brain structures.

#### Preparation of tissue extracts

The frozen brain samples were dropped in lysis buffer (10 µL of buffer/mg of tissue) containing 150 mM NaCl, 20 mM Tris Base, 1 mM EDTA, 1% Triton X-100, protease and phosphatase inhibitors (Roche). The samples were homogenized using a potter-elvehjem homogeneizer, and centrifuged at 13000×g for 10 min at 4°C. The protein concentration of the supernatant was determined by a Bradford assay (Biorad).

#### Antibodies

Primaries antibodies against phosphorylated forms of IGF-1 receptor (Tyr1135/1136), AKT (Ser473), GSK3β (Ser9) and p70S6K (Thr308) and total forms of AKT, GSK3β and IGF-1 receptor, and HRP-conjugated anti-rabbit or anti-mouse secondary antibodies were purchased from Cell Signalling Technology. Antibody against total form of p70S6K was purchased from Abcam, and α-tubulin from Sigma Aldrich. All experimental procedures were optimized for each antibody.

#### Immunoblotting

The samples were diluted in SDS-PAGE sample buffer (50 mM Tris-HCl, pH 6.8, 2% SDS, 10% glycerol, 5% β-mercaptoethanol and 0.1% bromophenol blue), heated 7 min at 95°C and resolved on 7.5% SDS polyacrylamide gels. The proteins were transferred to a 0.2 µm nitrocellulose membrane. The membrane was blocked with 5% non-fat dry milk in Tris-buffered saline Tween (20 mM Tris–HCl, pH 7.6; 150 mM NaCl, and 0.5% Tween 20) at room temperature. The same membrane was processed in three sequential steps: incubation with (i) the antibody against phosphorylated form of protein; (ii) antibody against the total form; and (iii) antibody against α-tubulin. Blots were firstly incubated overnight at 4°C with anti-P-IGF-1 receptor, anti-P-AKT, anti-P-GSK3β or anti-P-p70S6K antibodies. For detection, the membranes were incubated with HRP-conjugated anti-rabbit secondary antibody at room temperature. Immunoreactivity was detected using enhanced chemiluminescence (PerkinElmer) and detection was carried out on hyperfilms Biomax MR (Amersham). Blots were stripped with Western Re-probe buffer (Agro-Bio). The efficiency of the stripping was tested by incubation with the secondary antibodies and a revelation by chemiluminescent substrate. Blots were then re-probed with anti-IGF-1 receptor, anti-AKT, anti-GSK3β or anti-p70S6K antibodies. Blots were incubated with HRP-conjugated anti-rabbit secondary antibody and visualized as described above. Finally, blots were incubated overnight at 4°C with anti-α-tubulin primary antibody and thereafter with HRP-conjugated anti-mouse secondary antibody. The signals were revealed as above to check that equal amounts of proteins are loaded on the gel.

#### Densitometric analysis

All blots were scanned and densitometric analysis was conducted using GS-800 Imaging densitometer and QuantityOne Software (Biorad). Phosphospecific signal was normalized to the total signal to estimate the ratio of activated marker. Total signal was normalized to the α-tubulin signal, which was used as an internal control, to estimate protein expression.

### IGFBPs analysis

The expression of IGFBP3, IGFBP5 and the co-expression of IGF1 with these binding proteins were performed on plasma samples (n = 8 per group).

#### Albumin and IgG depletion

In order to evaluate the effect of HU on plasma IGF-1 environment, IGFBP3 and IGFBP5 were quantified on plasma samples depleted from albumin and IgG (ProteoPrep Blue Albumin and IgG Depletion kit, Sigma Aldrich). In a first step, 75 µL of plasma were added into depletion column, incubated for 10 min at room temperature and centrifuged at 10000×g for 60 s. The procedure was performed twice to improve albumin depletion by reapplying the eluate on the column. In a second step, remaining unbound proteins from the spin column were washed 2 times with equilibration buffer by 60 s centrifugation and pooled with the eluate obtained in the first step. The total fractions were then stored at −20°C for further experiments.

#### Western blot analysis

Depleted plasma samples were separated on 15% SDS polyacrylamide gels and analyzed by western blot as previously described, using antibodies against IGFBP3 (Abnova) and IGFBP5 (Abcam).

In order to evaluate IGF-1 bioavailability, co-immunoprecipitations were performed on 900 µg of depleted plasma samples. Each sample was first pre-cleared with protein G coupled on Magnetic Beads (Millipore). Non-retained samples were then incubated overnight with IGF-1 primary antibody (Abnova). Protein G coupled on magnetic beads was then added at a final dilution of 1/5 (v/v) for 1h30 at room temperature. Following this incubation, beads were washed 4 times with PBS 0.05% Tween and were finally resuspended in Laemmli buffer and boiled at 95°C for 10 min. Magnetic beads were carefully discarded and the remaining fraction was separated on 15% SDS polyacrylamide gels and analyzed by western blot using antibodies against IGFBPs.

#### Densotimetric analysis

For IGFBP3 and IGFBP5, total protein signals were normalized to ponceau S densitometric signal. For co-immunoprecipitation experiments, the quantification of IGF-1 bound to IGFBPs was normalized to the total form of IGFBP3 and IGFBP5.

### Statistical analyses

Results are presented as mean ± SEM. Normality was evaluated by Kolmogorov-Smirnov test. C and HU14 groups were compared by Student *t*-test when groups were normal or Mann-Whitney *t*-test otherwise. When the groups had significantly different variances, a *t*-test with Welch's correction was performed. Variances of IGF-1 plasma were compared by Barlett's test. The relationship between plasma and tissue levels of IGF-1 was assessed using the Pearson correlation coefficient. A p-value of less than 0.05 was chosen as the significance level for all statistical analyses.

## Results

### IGF-1 measurement in cerebral tissue and plasma

**Figure 1:**
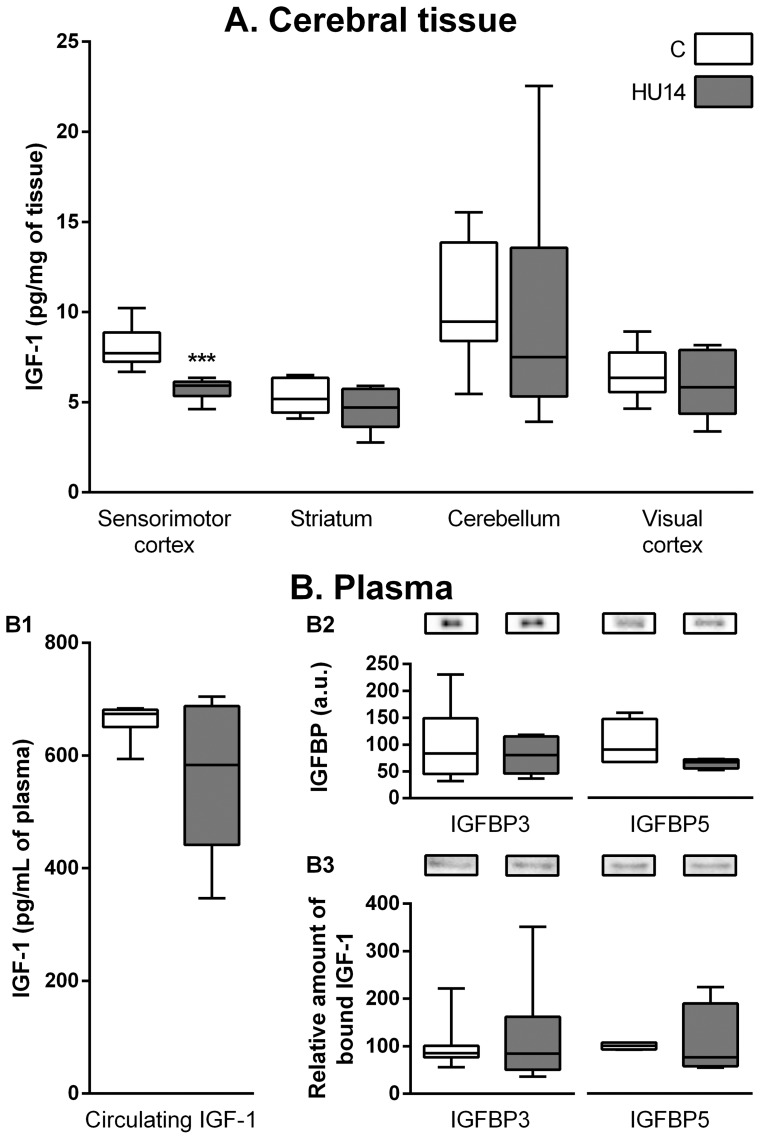
Effect of HU on IGF-1 level. (A) IGF-1 level in cerebral tissues, determined by ELISA, in control (C) and HU14 groups. (B) IGF-1 measurement in plasma. B1: Circulating IGF-1 determined by ELISA. B2: Total IGFBP3 (left) and IGFBP5 (right) expressed with respect to total protein level. B3: Quantification of IGF-1 bound to IGFBP3 (left) or IGFBP5 (right), expressed as the ratio between bounded IGF-1 and total IGFBPs, and representative western blot showing IGFBPs after IGF-1 immunoprecipitation. Box and whisker plots show the median (horizontal bar), quartiles (box), and extreme values (whiskers). *** p<0.001 with respect to C value.

We quantified IGF-1 in the sensorimotor cortex, the striatum and the cerebellum, and in one structure used as internal control (visual cortex) (Fig. 1A). In C rats, IGF-1 level was between 5 and 10 pg/mg in the different structures. After HU, IGF-1 level was decreased in the sensorimotor cortex (−28%; p<0.001). Levels were unchanged in striatum and cerebellum, and in control structure (visual cortex). It should be mentioned that values were homogenous in the C group whereas they were more scattered in HU14 group, except for sensorimotor cortex.

Although IGF-1 can be produced by the brain, the very low expression of IGF1 mRNA and the wide expression of IGF receptors in the adult mammalian brain suggest that the main source of IGF-1 in the brain corresponds to circulating IGF-1 [12]. Thus, we wanted to determine whether plasma level was affected by HU. The IGF-1 plasma level was 660±12 pg/mL in C rats (Fig. 1B1). It was reduced in HU rats (545±50 pg/mL), although not significantly (p = 0.06), due to a high individual variability between samples in this group (coefficient of variation: 24% in HU14 rats *vs*. 5% in C ones, Barlett's test: p<0.01).

Next, we determined whether changes in IGFBP levels could be responsible for the decrease in IGF-1 levels observed in the brain, since it has been shown that IGF-1 entry within the brain is decreased in presence of IGFBP3 [18]. No variations were found after HU, neither in the total IGFBP level (Fig. 1B2), nor in the relative quantity of IGF-1 bound to IGFBP3 or IGFBP5 (Fig. 1B3).

IGF-1 enters into the brain through the blood brain barrier. In order to determine whether the decrease in IGF-1 cerebral level was directly related to the decrease in plasmatic one, we performed a correlation analysis. Tissue level was plotted against plasma level in individual rats (Table 1). Pearson correlation coefficient indicated no relationship for both groups and for the different structures.

**Table 1:** Regression analysis performed between IGF-1 level in different cerebral tissues and plasma.

	Control		HU14	
	Slope	r^2^	Slope	r^2^
**Sensorimotor cortex**	0.02±0.02	0.14	0.00±0.00	0.12
**Striatum**	0.02±0.01	0.53	0.01±0.01	0.71
**Cerebellum**	−0.06±0.05	0.29	−0.02±0.04	0.15
**Visual cortex**	0.02±0.03	0.06	0.01±0.00	0.38

r^2^ is the coefficient of determination. Data are mean ± SEM.

### Phosphorylation and expression level of IGF-1 receptor

**Figure 2:**
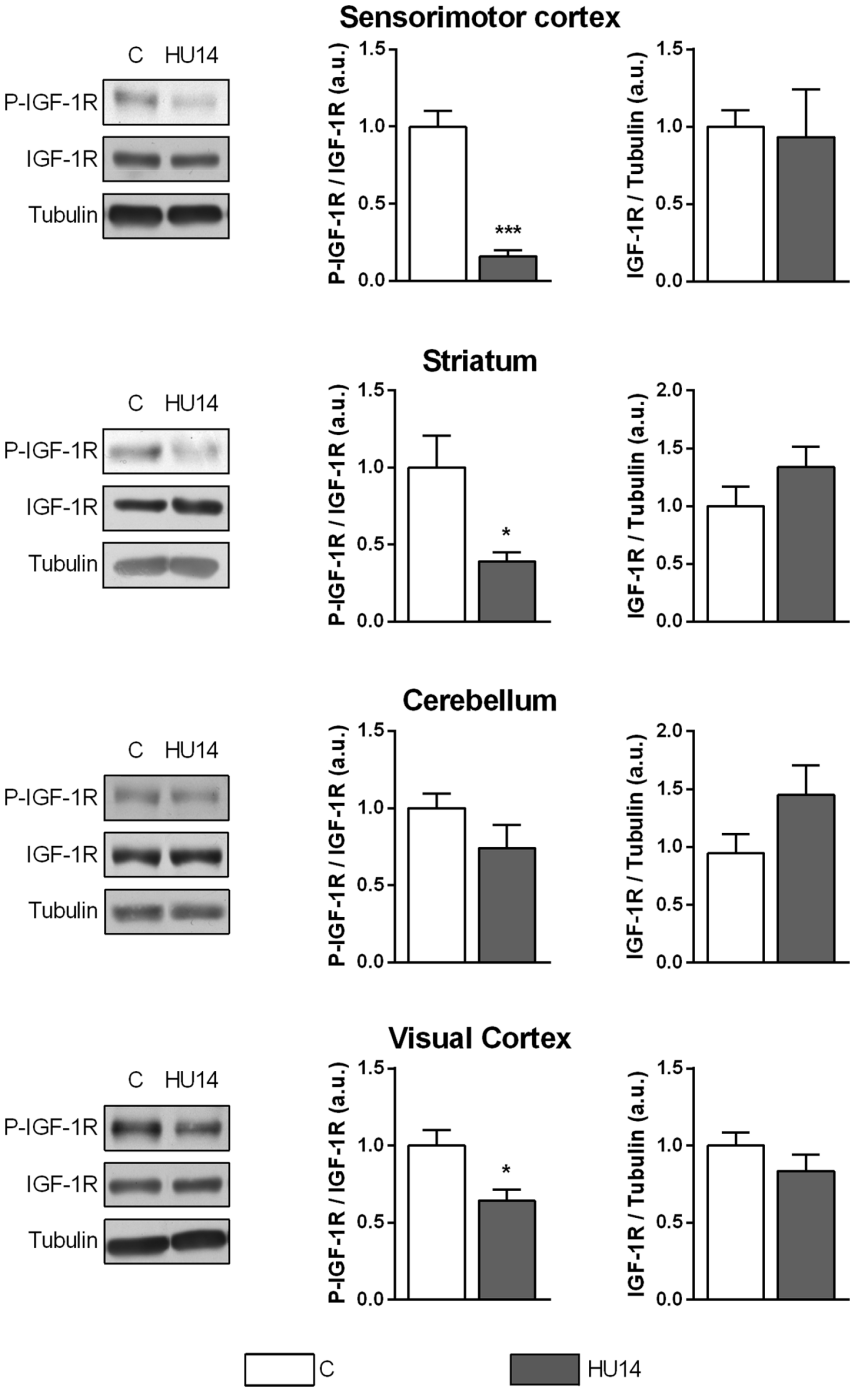
Phosphorylation and expression level of IGF-1R in brain. Left: representative western blot showing P-IGF-1R and total IGF-1R immunoreactivity in control (C) or HU14 groups. Middle: level of phosphorylation of IGF-1R, expressed as the ratio between P-IGF-1R and IGF-1R, in C and HU14 groups. * and *** p<0.05 and p<0.001 respectively, with respect to C group. Right: expression of IGF-1R, corresponding to the ratio between IGF-1R and α-tubulin). Values are mean ± SEM.

IGF-1 exerts its function by binding to and activating its specific receptor (IGF-1R). Thus, we next evaluated the phosphorylation level of IGF-1R in the brain, and its expression level by western blot (Fig. 2). IGF-1 receptor phosphorylation level was expressed as the ratio between P-IGF-1R and IGF-1R levels. This level decreased in sensorimotor cortex (−82%, p<0.001), striatum (−60%, p<0.01) and visual cortex (−30%, p<0.05) but not in the cerebellum. This decrease can be explained by a decrease in IGF-1R phosphorylation since IGF-1R expression level, normalized to α-tubulin, was unchanged.

### Phosphorylation and expression level of PI3K-AKT pathway proteins

IGF-1R in brain is associated with PI3K-AKT signaling pathway. Phosphorylation and expression level of AKT, p70S6K and GSK3β proteins were evaluated in order to determine whether the decrease in IGF-1R phosphorylation after HU induced a modulation of this pathway.

**Figure 3:**
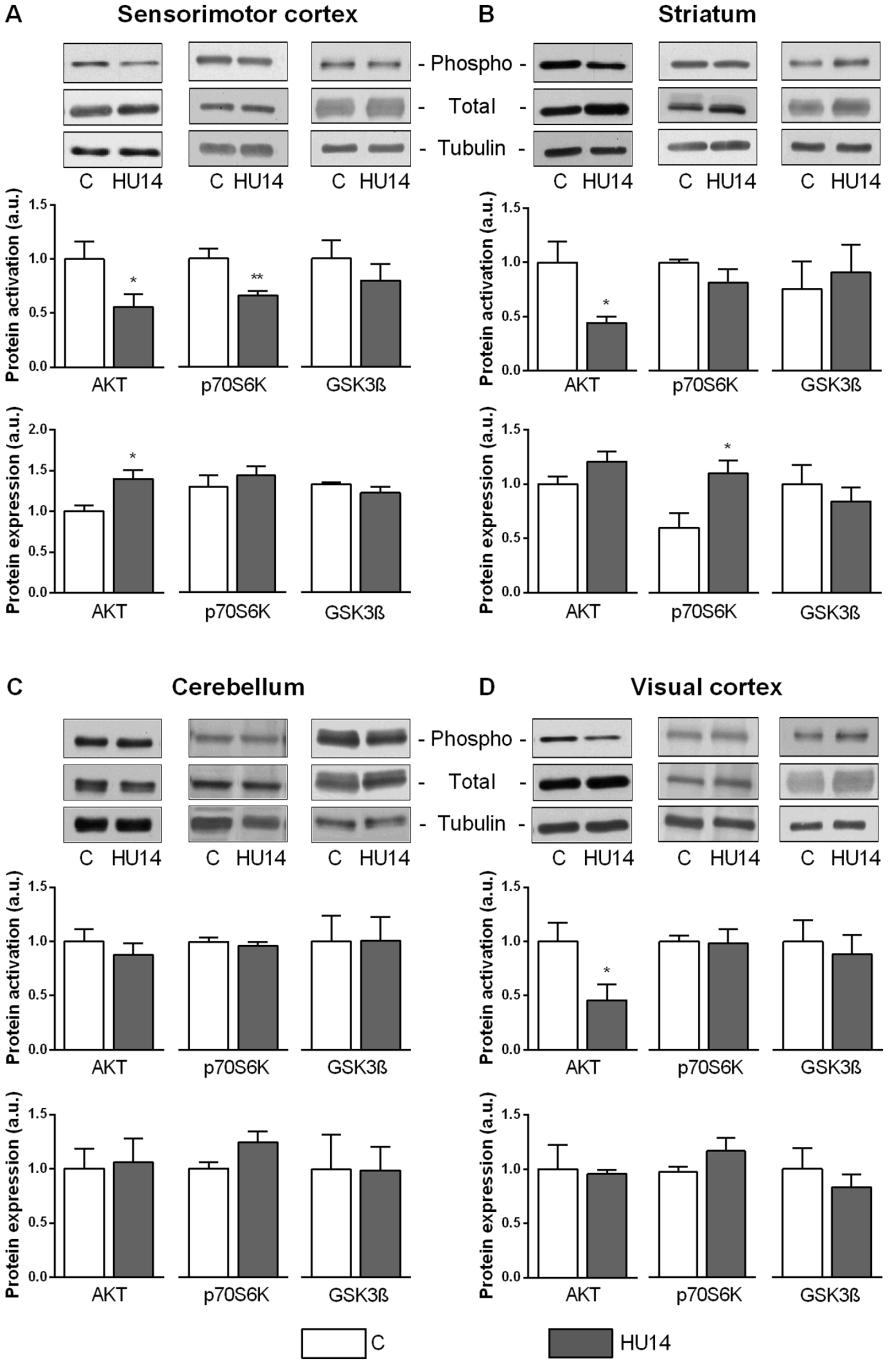
Phosphorylation and expression level of proteins of the PI3K-AKT pathway in brain. Representative western blot showing immunoreactivity for phosphorylated and total forms of AKT, p70S6K and GSK3β (above), phosphorylation level (middle), and expression level (below) in control or HU14 groups, in sensorimotor cortex (A), striatum (B), cerebellum (C) and visual cortex (D). *and ** p<0.05 and p<0.01 respectively with respect to C group. Values are mean ± SEM.

In the sensorimotor cortex (Fig. 3A), AKT phosphorylation level was decreased in HU rats (−45%, p<0.05) whereas AKT expression was increased (+40%, p<0.05). p70S6K and GSK3β are two downstream targets of AKT. The phosphorylation level was decreased for p70S6K (−37%, p<0.01) and remained constant for GSK3β. The expression level of these proteins was unchanged in HU14 group.

AKT phosphorylation also showed a decrease in the striatum (−56%, p<0.05), without any change in expression level (Fig. 3B). For p70S6K, no change was found in phosphorylation level, but at contrast with other structures, data revealed an increase in expression level (+84%, p<0.05). For GSK3β, neither phosphorylation level, nor expression one were affected by HU.

In the cerebellum (Fig. 3C), our results indicate no variation in phosphorylation or expression levels of the PI3K-AKT pathway members.

In the visual cortex (Fig. 3D), a decrease of AKT phosphorylation was observed (−54%, p<0.05). The expression level was unchanged. This reduction in AKT phosphorylation had no consequence on its target p70S6K, since no change was noticed in its phosphorylation or expression level.

## Discussion

We have investigated the impact of hypoactivity on IGF-1 and associated signaling pathway in structures implied in motor functions (sensorimotor cortex, striatum and cerebellum). We observed several changes in the visual cortex, suggesting that HU can induce an overall effect on the brain. However, the main changes were observed in somatosensory cortex and striatum, where HU leads to a decrease in IGF-1 level; this effect is correlated to a decrease in activation of its receptor and downstream targets (AKT and p70S6K, but not in GSK3β). Taken together, the present results suggest that a low physical activity might induce functional alterations of sensorimotor cortex through a decrease in IGF-1 pathway.

### HU has an effect on the whole brain

HU induces changes in tissue IGF-1 level, in activation of its specific receptor, and in its associated signaling pathway, in sensorimotor cortex and striatum, and with less intensity in the visual cortex. It suggests that HU can affect the whole brain. Ultrastructural changes similar to those described for somatosensory cortex have been described in the visual cortex of rats exposed to a 7-day or 14-day spaceflight [19]. HU reproduces the chronic weightless bearing, reduction in hindlimb movement and cephalic fluid shift observed in real microgravity. The large-scale action of HU on the brain can be the result of the cephalic fluid shift, which in turn produces, in primates as in rats, changes in intracranial blood volume, regional blood flow, vascular resistance capillary filtration, and even edema [20].

### Plasmatic IGF1 level tends to decrease in HU rats

Several investigators have found a correlation between exercise, plasma IGF-1 level, and secretion of growth hormone (GH), the main regulator of IGF-1 secretion by the liver. However, to our knowledge, the effects of a decrease in physical activity on plasma IGF-1 level have rarely been explored. Our results showing that IGF-1 serum level tended to decrease after a 14-day period of HU are in accordance with Perrien et al. [21].

The two relevant sources of IGF-1 in the periphery are the liver and the muscle. Thus, a muscle contribution to the reduction of the circulating blood levels should be considered. However, the expression of IGF-1 mRNA is unaffected in the soleus muscle of rats submitted to 14 days of HU [22] and of mice unloaded for 8 days [23], or in human vastus lateralis after 5 weeks of limb unloading [24]. These data suggest that the decrease in IGF-1 plasmatic level is mainly due to a lower synthesis within the liver.

The decrease in IGF-1 level might be due to the decrease in GH secretion induced by HU [25]. Besides, we cannot exclude that IGF-1 level varies in response to a stress induced by the unloading procedure since a chronic stress is known to decrease the IGF-1 level [26]. However, we found no change in the level of blood corticosterone in rats submitted to 14 days of HU (Figure S1).

### IGF-1 level is strongly decreased in sensorimotor cortex of HU rats

A decrease in tissue IGF-1 has already been demonstrated in the spinal cord of rats following unilateral hindlimb immobilization [27]. Our first hypothesis is that local synthesis of IGF-1 might be affected by hypoactivity. However, only small amounts of IGF-1 mRNA have been detected in the adult cerebral cortex and in the striatum [28], and the main origin of IGF-1 in the brain is peripheral. In adult, serum and brain IGF-1 levels are strongly dependent; for instance, a reduction in circulating IGF-1 using a viral vector approach decreases hippocampal IGF-1 protein levels [29]. Relative amount of IGF-1 mRNA is more than 100 times higher in the liver than in the brain [28], and approximately 95% of the IGF-I that acts on the brain is derived from the liver [30]. Thus, it is very likely that even a significant change in paracrine IGF-1 gene expression should be negligible with respect to the circulating (endocrine) IGF-1. However, the correlation analysis showed the absence of linkage between serum and brain tissue levels. One should take into account the blood brain barrier. Entry of IGF-1 from the plasmatic compartment into the brain can be achieved through the intervention of blood brain barrier and the blood cerebrospinal fluid barrier. Data indicate that the choroid plexus is the major route of entry of IGF-1 into the brain [31]. Exercise is known to increase brain uptake of IGF-1 [1], at least in part through the choroid plexus, since exercise increases the level of megalin, a transporter involved in IGF-1 transport into the brain [32]. However, whether disuse affects megalin level is unknown. A recent study showed that uptake of serum IGF-1 by the blood brain barrier is activity-dependent: neuronal activity elicited by sensory or behavioral stimulation increases IGF-1 entrance in activated regions but not in other parts of the brain [18]. In our study, HU is characterized by a reduced motor activity and by a deactivation of the cutaneous receptors located on the foot sole. Thus, we can hypothesize that the activity of neurons in the hindlimb cortical representation is reduced and in consequence, the permeability of blood brain barrier for IGF-1 should be decreased in the sensorimotor cortex.

IGF-1 level and activation of receptors was remarkably constant within the cerebellum. In control rats, cerebellum shows high levels of IGF-1. This is consistent with previous reports [33]. Local synthesis [28] and receptor density [34] are three times more abundant in the cerebellum than in the cerebral cortex or striatum. Given the well known implication of cerebellum in sensorimotor control, one would expect a decrease in tissue IGF-1 level after HU but we did not detect any variation in this structure. This result can be explained by the unloading protocol: suspending the rats from the ground at a 30° angle influences the head position as well as the neck angle. The activity of the cerebellum which receives information from the otolithic organs and neck proprioceptors [35] may be changed accordingly. Thus, a putative effect of hypoactivity could have been masked by the posture change induced by the HU procedure.

### HU does not affect the level of IGF-1 receptors, but decreases their activation in sensorimotor cortex

After binding to its receptor, IGF-1 induces the beta-subunit autophosphorylation of the receptor. We found no change in IGF-1 receptors following 2 weeks of disuse. This result is in accordance with Suliman et al. [27], who explored the regulation of IGF-1 receptor in the spinal cord. These authors found no change after a 2-wk period of immobilization, however, an upregulation of IGF-1 receptors was observed after a long-term immobilization (4 and 8 weeks). An increase has also been observed in the cerebellum of aged rats [36] and in the spinal cord of amyotrophic lateral sclerosis patients [37]. Taken together, these data suggest that a longer period of HU (i.e. >2 weeks) might also induce an upregulation of IGF-1 receptors. The latter can be viewed as a homeostatic mechanism to compensate for the reduction in IGF-1 level in brain tissue.

We also reported a decrease in receptor phosphorylation. It can be the consequence of the lower level of IGF-1 in tissue, and/or of a higher level of IGF binding proteins, internalization or desensibilisation of IGF-1R, or dephosphorylation by action of phosphatases.

The role of striatum in the planning and modulation of movement pathways is well documented [38]. A reduction of excitatory cortical input in the striatum during HU will in turn affect striatal cells discharge. As expected, HU decreases receptor activation. However, effect on the signaling pathway is only moderate since basal ganglia are not devoted to strictly motor functions, but are also involved in cognitive functions.

### Consequences of IGF-1 receptor activation and functional implications of the decrease in IGF-1 level

IGF-1 is involved in dendritic structure and synaptic connectivity. In postnatal rat slice cultures of somatosensory cortex, IGF-1-treatment leads to increased dendritic branching of pyramidal neurons [39], whereas application of an IGF-1 antibody prevents exercise-induced increase of dendritic spines within the hippocampus [40]. This effect is mediated by the PI3K/AKT/mTOR/p70S6K cascade [41]. As a consequence, the decrease in p70S6K phosphorylation observed in the sensorimotor cortex of HU rats should have induced a decrease in spine density and in dendritic length. In a recent study [8], we observed a decrease in the diameter of dendritic branches and in spine length, which is consistent with a role of IGF-1 in dendritic growth. However, we also reported an increase in the density of dendritic spines. Such an increase in spine density is puzzling in view of the present results. However, dendritic spines maintenance can be impacted by other substances, such as NGF or BDNF, which expression are increased in the sensorimotor cortex of HU rats [42].

IGF-1 is also known to modulate cell activity by regulating calcium entry into cells. IGF-1 stimulates calcium entry by translocating a specific channel (“growth-factor-regulated channel”) to the plasma membrane, via a PI3K dependent mechanism [43]. In addition, Blair and Marshall [44] provided evidence for a large increase in calcium channel currents in cerebellar granule neurons in response to IGF-1, mediated by PI3K. In the same way, IGF-1 enhances Ca^2+^ channel currents in pyramidal neurons of motor cortex in the rat [45], but the intracellular pathway mediating this effect is unknown. Thus, a decrease in IGF-1 level could be associated to a decrease in calcium entry, and might affect neurotransmitter release and firing behavior of pyramidal neurons. It could be the substrate of the decrease in neuronal excitability observed in deep layers of the motor cortex of HU rats [46]. At the opposite to layer V neurons, the activity is increased in layer IV within the sensorimotor cortex [38], [47]. The fact that IGF-1 receptors are mainly located in supra- and infragranular layers, but more rarely in granular one ([34] and Figure S2) sustains the hypothesis of a participation of IGF-1 to a Hebbian mechanism of activity-dependent regulation of neuronal excitability, leading to a hypoexcitability of corticospinal fibers.

### Conclusion

IGF-1 is often considered as the central node of a complex system of homeostatic regulation [12], and as the mediator of exercise-induced benefits on the brain [1], [3]. However, studies explored mainly the effect of exercise on cognition and hippocampus, more rarely the consequences of physical activity on the sensorimotor system. Yet, the functional repercussions of the changes in IGF-1 level and associated signaling pathway in structures involved in sensorimotor control should be determined. In the dorsal column nuclei, intracarotid injection of IGF-1 increases the response of neurons to a peripheral stimulus, and increases the extension of receptive fields [48]. These effects are similar to those observed in the cerebral cortex of HU rats [47], [49], where IGF-1 level is dramatically lowered. In this perspective, the relationships between IGF-1 and the somatosensory cortical representation of the body merits further exploration. A better knowledge of the role of IGF-1 in activity-dependent plasticity might allow identifying new target for therapeutic tools for immobilized patients.

## Supporting Information

Figure S1 **Corticosterone level is transitorily increased within the first three days of unloading, and recovered normal values after 7 days.** The same observation has also been made by others [16]. Thus, the decrease in IGF-1 plasmatic level cannot be attributed to a response to stress. The level of blood corticosterone was determined in C rats, and in rats submitted to 3, 7 or 14 days of HU. After rat decapitation, a blood sample was taken in a tube containing EDTA (Becton Dickinson) to prevent coagulation and placed in ice. Plasma was collected after centrifugation (3000×g for 15 min), froze in liquid nitrogen and store at −80°C. Corticosterone levels were evaluated with an ELISA kit (Corticosterone EIA, Immunodiagnostic systems). All procedure (plasma sample, dilution solution, measurement and reagent preparation) was performed according to the instruction given by the constructor protocol. Data are mean ± SEM. * p<0.05 with respect to Control value, # p<0.05 with respect to HU value.
(TIF)

Figure S2 **Cells immunoreactive for IGF-1 were mainly encountered in layers II/III and V/VI, and more rarely in granular layer.** Rats were deeply anesthetized with pentobarbital sodium (60 mg/kg, i.p.) and perfused transcardially with cold 0.9% normal saline followed by 300 ml of 0.1 M phosphate buffer (PB) containing 4% paraformaldehyde and 0.1% glutaraldehyde. The brain was immediately removed and post-fixed in the same fixative for 4 h and cryoprotected in 30% sucrose for 24 h at 4°C. The brain was serially sectioned in a coronal plane into 40-µm slices with microtome. Sections were then incubated in 1% bovine serum in the presence of 0.2% Triton X-100 for 1 h at room temperature, followed by IGF-1R antibody (Cell Signaling Technology), overnight at 4°C. After rinsing, they were incubated with a goat Anti-Rabbit IgG fluorescein conjugated secondary antibody (Abcam) in 1% bovine serum for 1 h. The sections were rinsed, mounted in Vectashield medium (Vector Laboratories), and examined under a fluorescent microscope.
(TIF)
