# Supplementary figures and images for: Hypoactivity Affects IGF-1 Level and PI3K/AKT Signaling Pathway in Cerebral Structures Implied in Motor Control
Source: PLoS One. 2014 Sep 16;9(9):e107631. doi: 10.1371/journal.pone.0107631 (PMC4166665; doi:10.1371/journal.pone.0107631)

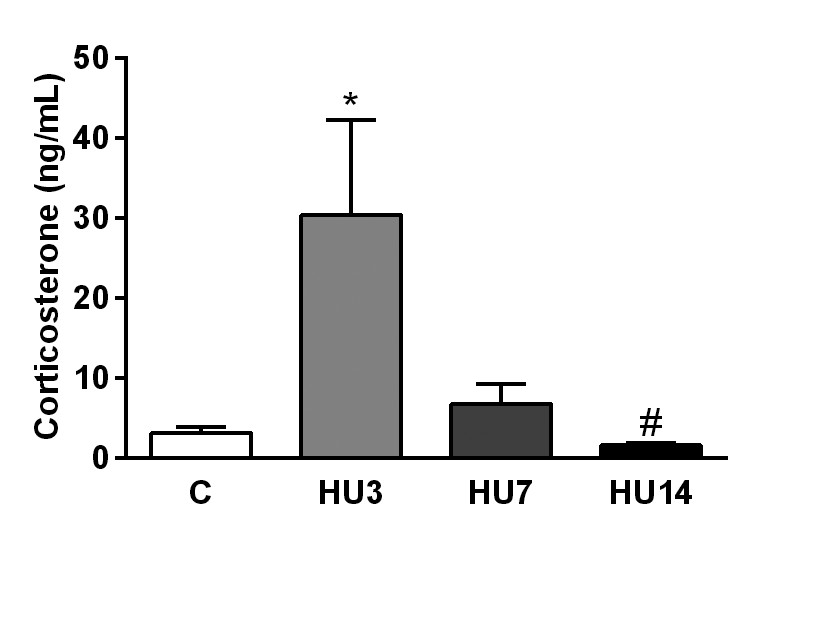

Supplement: Figure S1 — Corticosterone level is transitorily increased within the first three days of unloading, and recovered normal values after 7 days. The same observation has also been made by others [16]. Thus, the decrease in IGF-1 plasmatic level cannot be attributed to a response to stress. The level of blood corticosterone was determined in C rats, and in rats submitted to 3, 7 or 14 days of HU. After rat decapitation, a blood sample was taken in a tube containing EDTA (Becton Dickinson) to prevent coagulation and placed in ice. Plasma was collected after centrifugation (3000×g for 15 min), froze in liquid nitrogen and store at −80°C. Corticosterone levels were evaluated with an ELISA kit (Corticosterone EIA, Immunodiagnostic systems). All procedure (plasma sample, dilution solution, measurement and reagent preparation) was performed according to the instruction given by the constructor protocol. Data are mean ± SEM. * p<0.05 with respect to Control value, # p<0.05 with respect to HU value. (TIF) [file pone.0107631.s001.tif]

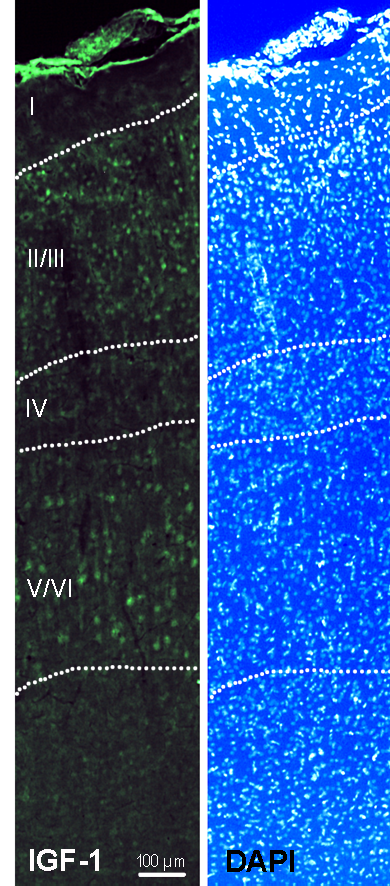

Supplement: Figure S2 — Cells immunoreactive for IGF-1 were mainly encountered in layers II/III and V/VI, and more rarely in granular layer. Rats were deeply anesthetized with pentobarbital sodium (60 mg/kg, i.p.) and perfused transcardially with cold 0.9% normal saline followed by 300 ml of 0.1 M phosphate buffer (PB) containing 4% paraformaldehyde and 0.1% glutaraldehyde. The brain was immediately removed and post-fixed in the same fixative for 4 h and cryoprotected in 30% sucrose for 24 h at 4°C. The brain was serially sectioned in a coronal plane into 40-µm slices with microtome. Sections were then incubated in 1% bovine serum in the presence of 0.2% Triton X-100 for 1 h at room temperature, followed by IGF-1R antibody (Cell Signaling Technology), overnight at 4°C. After rinsing, they were incubated with a goat Anti-Rabbit IgG fluorescein conjugated secondary antibody (Abcam) in 1% bovine serum for 1 h. The sections were rinsed, mounted in Vectashield medium (Vector Laboratories), and examined under a fluorescent microscope. (TIF) [file pone.0107631.s002.tif]
